# Evidence of long-term NAO influence on East-Central Europe winter precipitation from a guano-derived δ^15^N record

**DOI:** 10.1038/s41598-017-14488-5

**Published:** 2017-10-26

**Authors:** Daniel M. Cleary, Jonathan G. Wynn, Monica Ionita, Ferenc L. Forray, Bogdan P. Onac

**Affiliations:** 1 School of Geosciences, University of South Florida, 4202 E. Fowler Ave, NES 107, Tampa, FL 33620 USA; 2 Paleoclimate Dynamics Group, Alfred-Wegener-Institute for Polar and Marine Research, Bussestrasse 24, Bremerhaven, D-27570 Germany; 3 MARUM, Center for Marine Environmental Sciences, University of Bremen, Bremen, Germany; 4 Department of Geology, Babeș-Bolyai University, Kogălniceanu 1, 400084 Cluj-Napoca Romania; 5 Emil Racoviță Institute of Speleology, Romanian Academy, Clinicilor 5, Cluj-Napoca 400006 Romania; 6 Present Address: National Science Foundation, 4201 Wilson Blvd., Arlington, VA 22230 USA

## Abstract

Currently there is a scarcity of paleo-records related to the North Atlantic Oscillation (NAO), particularly in East-Central Europe (ECE). Here we report δ^15^N analysis of guano from a cave in NW Romania with the intent of reconstructing past variation in ECE hydroclimate and examine NAO impacts on winter precipitation. We argue that the δ^15^N values of guano indicate that the nitrogen cycle is hydrologically controlled and the δ^15^N values likely reflect winter precipitation related to nitrogen mineralization prior to the growing season. Drier conditions indicated by δ^15^N values at AD 1848–1852 and AD 1880–1930 correspond to the positive phase of the NAO. The increased frequency of negative phases of the NAO between AD 1940–1975 is contemporaneous with higher δ^15^N values (wetter conditions). A 4‰ decrease in δ^15^N values at the end of the 1970’s corresponds to a strong reduction in precipitation associated with a shift from negative to positive phase of the NAO. Using the relationship between NAO index and δ^15^N values in guano for the instrumental period, we reconstructed NAO-like phases back to AD 1650. Our results advocate that δ^15^N values of guano offer a proxy of the NAO conditions in the more distant past, helping assess its predictability.

## Introduction

**Figure 1:**
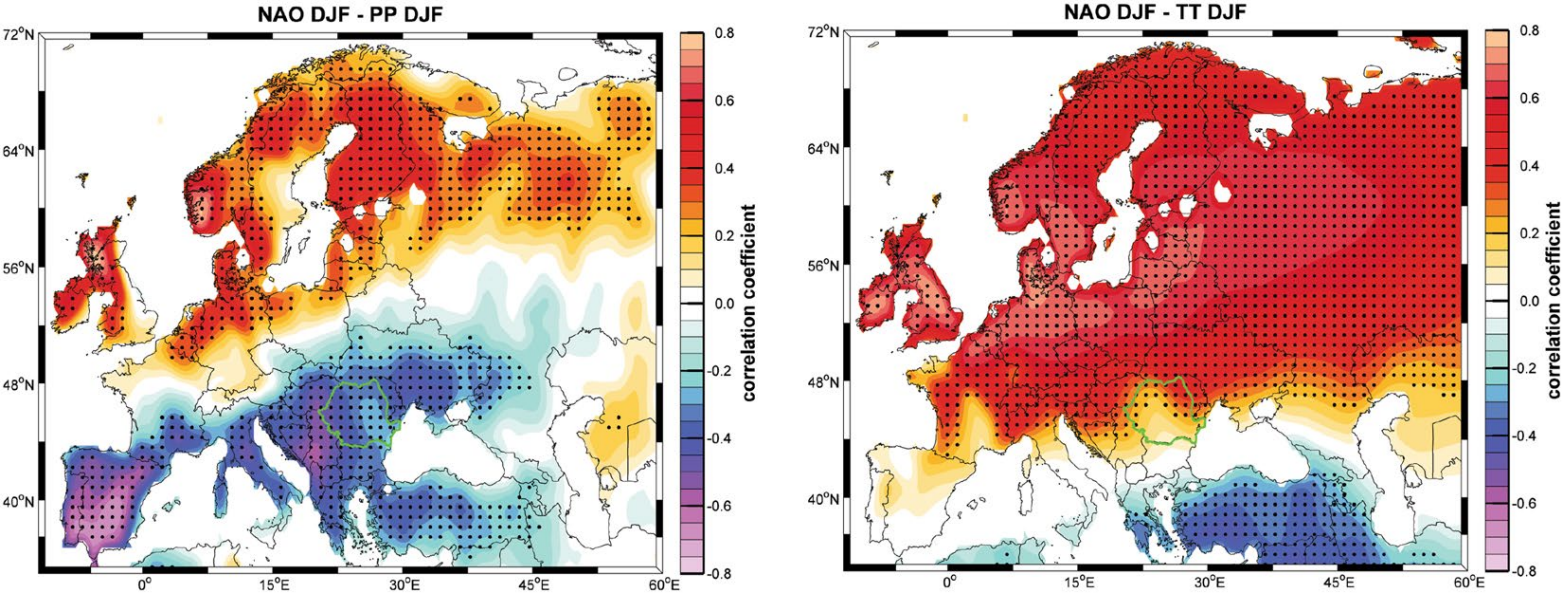
Correlation map between (**a**) winter (DJF) NAO index and winter precipitation (PP), and (**b**) temperature (TT) derived from the CRU TS3.24.01 data set^54^. Green contours outline Romania. Dotted areas indicate correlation coefficients significant at 95% significance level on a standard t-test. The figure was produced using Matlab 2014b (http://de.mathworks.com/products/new_products/release2014b.html).

Global atmospheric circulation has a number of preferred patterns of variability, all of which have expressions in surface climate. Regional climates may vary out of phase, due to the action of such teleconnections, which modulate the location and strength of the storm tracks and poleward fluxes of heat, moisture and momentum^1–3^. Over Europe, the strongest influence is given by the North Atlantic Oscillation (NAO)^4–6^. NAO has a substantial effect on the European climate by modulating the position of North Atlantic storm tracks, which in turn control short and long-term changes in precipitation and temperature. When NAO is in its positive phase (*e.g*., a stronger than normal Azores High and a deeper than normal Icelandic Low), winter storm tracks are deflected northward resulting in wet winters over the northern part of Europe (positive correlation between winter NAO index and winter precipitation) and dry winters over the southern and eastern part of Europe (negative correlation between winter NAO index and winter precipitation) (Fig. 1). During the negative phase of NAO, storm tracks are shifted southwards, thus bringing wet and mild winters over the southern and eastern parts of Europe and dry winters over the northern part of Europe.

At a regional scale, the Carpathian Mountains have a strong influence on NAO related precipitation and temperature anomalies in Romania^5^. Due to orographic effect of the Carpathians, the winter NAO related signal is stronger in the northwestern part of the country^7,8^ where Măgurici Cave (hereafter MC) is located (see Supplementary Fig. S1). While studies have demonstrated that the NAO is an important factor in winter climate variability in East-Central Europe (ECE)^8–10^, except a recent study reconstructing winter air temperature and moisture source^11^, there is a lack of paleo-records of the NAO in ECE. Furthermore, to place recent NAO variability in a long-term context, there is a pressing need to develop longer records.

Here we use coupled carbon and nitrogen isotope ratio data (δ^13^C and δ^15^N values) of a 286 cm guano core from MC in ECE, in an attempt to characterize the hydroclimate influence on the N-cycle since the latest part of the Little Ice Age (LIA; AD 1650 to 1850) and up to AD 2012. We first compare these data to the December-January-February (DJF) NAO index that represents the strength of the system during the winter months. Then we use DJF meteorological records and a ECE reconstructed precipitation series from Pauling *et al*.^12^ that directly and indirectly relate to the NAO to further examine the impact of changes in vegetation and hydrology of ECE on the isotopic composition of guano.

## Guano and Climate

Bat guano is primarily composed of loose organic material, such as insect chitin, with a geochemistry characterized by an abundance of transition metals^13,14^. Although there are numerous organic compounds in guano, chitin is the most abundant in MC deposit. Therefore, measured δ^15^N values reflect chitin derived N, while the contribution of other N sources to bulk guano is likely insignificant. If bats are not changing their roost sites, guano can accumulate as thick deposits (3 m or more) over long time periods (centuries to thousands of years)^13,15^. Depending on the morphology and hydrology of each cave system, flooding of underground passages may cause guano deposits to be interbedded with clay and silt layers. Such circumstances offer additional information with respect to local cave environmental changes while guano was being deposited^16,17^.

Bat guano may provide an ideal record of past vegetation because the δ^13^C value of plants in the region is transferred from plant to insect to bat and ultimately recorded in guano. The δ^13^C values of foliage are distinct between the two main photosynthetic pathways (C_3_ and C_4_)^18^. Additional variation in δ^13^C values occurs within C_3_ plants in response to the water use efficiency of photosynthesis^19^. The preservation of these values within guano provides new and critical information on the changing vegetational assemblage through time^20–25^. Although more complex, recent studies have demonstrated that δ^15^N values in bulk guano can be interpreted as an integrator of the nitrogen cycle (N-cycle)^26,27^. When nitrogen is a limiting nutrient, nitrogen is conserved and as a result less nitrogen is lost and δ^15^N values decrease (*i.e*., a relatively closed N-cycle)^28,29^. A relatively open cycle results in high δ^15^N values under the opposing conditions. When it can be demonstrated that a climatic influence controls whether the N-cycle is open or closed, δ^15^N values can also be used as a paleoclimatic proxy^26^. Since the NAO has a strong influence on precipitation and temperature it is possible that the nitrogen isotopic composition of cave bat guano, which has already been shown to reflect changes in water availability could provide insight into the influence of the NAO beyond the historical record.

## Results and Discussions

The nitrogen isotopic composition of bat guano may be affected by diagenesis after deposition via processes of ammonia volatilization or denitrification^13,30^. The conditions under which alteration may have been significant are in part reflected in variation of %N along the profile (see Cleary *et al*.^26^). The %N in the MC core (see Supplementary Dataset 1) shows little deviation from near surface values (~11.3%) to the lower most portion of the core (mean = 10.6%). Therefore, the increasing trend in δ^15^N values between AD 1650 and AD 1980 (see Supplementary Fig. S2) is likely unrelated to diagenesis and can be interpreted as primary variation, considered here as a proxy of the NAO variability. The subsequent interval of lower δ^15^N values (7.5 to 9.5‰; AD 1980 to 2012) represents a change in local N-cycle (see discussion below) as opposed to diagenetic processes.

It is difficult to elucidate past δ^15^N values at any stage of the food web (plant-insect-bat-guano), however it is possible to interpret δ^15^N as an integrator of the N-cycle^28^. The isotopic fractionations, N pool mixing, and N gains/losses produce the δ^15^N value of the system (soil, biomass, consumers). Therefore this resulting value integrates these processes and can be used to interpret the state of the N-cycle. The fractionations occurring during the metabolic processes within bats and insects as nitrogen is transferred from plant to guano remain fixed through time. Since these fractionations follow conservative pathways, variation in the nitrogen isotopic composition of guano can ultimately be related to changes that occur in the soil inorganic nitrogen reservoir from which plants access nitrate and ammonia^28,29^. Such changes have been connected to the state of the N cycle (open: more nitrogen loss and higher δ^15^N values; closed: less nitrogen loss and lower δ^15^N values)^29^ and attributed to hydrological influence on the state of the N-cycle^31–33^.

Cleary *et al*.^26^ suggested variation in δ^15^N values of guano (ultimately related to those of foliage) are strongly correlated to instrumental record of winter precipitation. This may result from a lag between the preservation of δ^15^N values in foliage during the growth phase (spring-early summer), which reflect soil N conditions from months immediately prior to growth (late fall-winter). In temperate forests, the maximum plant-available N occurs just prior to the onset of the growing season due to limited plant uptake of N that has been produced largely by microbial mineralization in months prior^34^. Although there may be significant wet deposition of N during the spring and summer months, this flux into terrestrial ecosystems is on average lower than that produced via mineralization^35,36^. This pool of plant-available N is soluble; thus during the winter season it is very sensitive to leaching processes, which is in turn driven by winter climatic conditions such as snow melt and the type of winter precipitation (snow vs. rain) when temperatures that straddle the threshold of freezing^37,38^. Since the state of the N-cycle is controlled by the amount of N in the system, we infer that any change in the N-cycle is in response to the impact of winter hydroclimate on leaching processes. During the subsequent growing season when plants begin to access the soil N-pool, the state of the N-cycle established by the amount of leaching in the winter is then recorded in the new foliage. Increased leaching would reflect a more open N-cycle and result in higher δ^15^N values^39^ at each level of the food chain and ultimately guano. Therefore, although bats forage in the summer months, nitrogen delivered to the soil via spring/summer precipitation will likely not influence the δ^15^N values of guano.

Cleary *et al*.^26^ compared δ^15^N values of bat guano to corresponding δ^13^C values and to meteorological data from a nearby weather station in the Mada region (Metaliferi Mountains, W. Romania), and interpreted that water availability is a primary control of δ^15^N values of guano. Given the well-documented relationship of δ^13^C values of leaf tissues to water-use efficiency^19,40^ the carbon isotopic composition of MC guano (−26.5 to −21‰) suggests variation within the C_3_ pathway that may ultimately be related to changes in water availability. Based on these observations, we test a hypothesis for a hydrological connection between δ^13^C and δ^15^N values of guano by examining the correlation between each proxy in the Măgurici Cave guano record. Excluding two outliers, the resulting statistical analysis indicates that the δ^15^N and δ^13^C values in the MC record between AD 1800 and 2012 show a negative correlation (p-value = <0.001; R^2^ = 0.62; n = 105; see Supplementary Fig. S2a). This suggests some dependence of the N-cycle of the bats foraging area on water availability, as the latter can be interpreted as the primary control on δ^13^C values of C_3_ vegetation. The relationship between δ^15^N and water availability is in contrast to other studies^32,33,41^, however, is consistent with results obtained from modern guano^25^ and precipitation in NW Romania.

Although both δ^15^N and δ^13^C values of guano reflect hydrological conditions, it is likely that they are recording different seasonal influences on water availability. While δ^13^C values of foliage are related to water stress during photosynthesis (spring/summer), δ^15^N values of guano are related to the winter precipitation influence on leaching. Therefore, correlation between δ^13^C values and δ^15^N values is ultimately the result of the winter precipitation (control of N-cycle) contributing to the degree of water stress in the spring/summer (control of δ^13^C). Consequently, if there is an influence of the NAO on precipitation in ECE, the nitrogen isotopic composition of guano should retain this signal more accurately than δ^13^C values. In accordance, hereafter we focus our interpretations of δ^15^N values of guano on reconstructing DJF precipitation.

**Figure 2:**
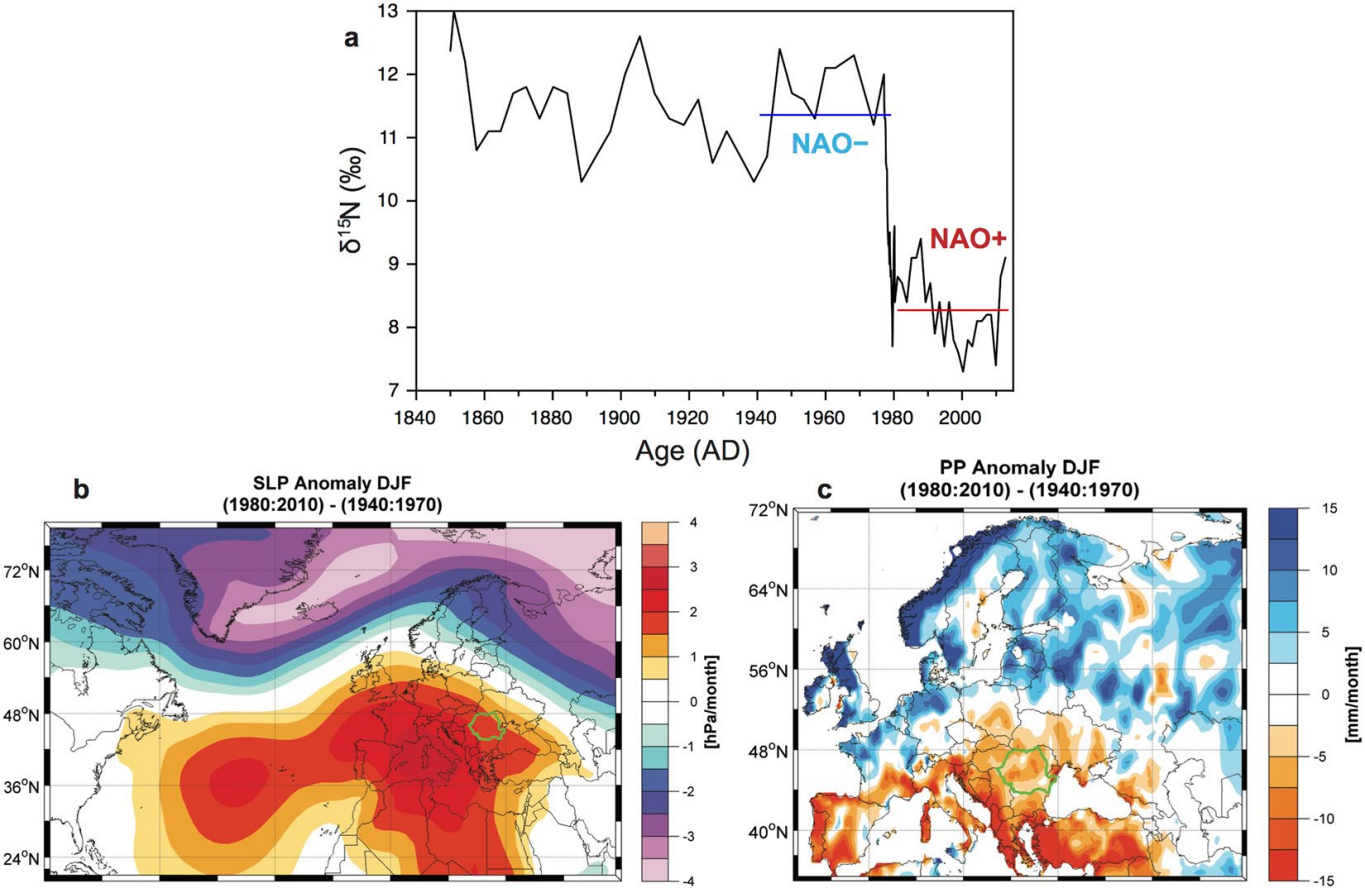
(**a**) δ^15^N of MC guano from 1850 to 2012 (see text for details). (**b**) The difference in the Sea Level Pressure between 1940 and 1970 (pre-shift) and the period 1980–2010 (post-shift) based on data from NCEP/NCAR Reanalysis^55^. (**c**) As in (**b**) but for winter precipitation derived from CRU TS3.24.01 data set^54^. The figures were produced using (**a**) pro Fit 7.0 (http://www.quansoft.com) and (**b,c**) Matlab 2014b (http://de.mathworks.com/products/new_products/release2014b.html), respectively. The digital version was generated using Adobe Illustrator CC17 (https://www.adobe.com/products/illustrator.html).

One of the most striking features of our δ^15^N reconstruction is the abrupt decrease (~4‰) at the end of 1970’s (Fig. 2a) representing a shift of the NAO to a strong positive phase after the mid-1970s. This swing corresponds to regional climatic effects such as milder and wetter winters in northern Europe^42^, a reduced discharge of the Danube River^43^, and decreasing winter precipitation over Romania^44^. This shift at the end of 1970’s is clearly observed in the sea level pressure (SLP) and precipitation patterns (Fig. 2). The difference map in the SLP field between 1940–1970 and 1980–2010 is indicative of a period characterized by an increased frequency of positive NAO phases after the 1970s (Fig. 2b), which is associated with a strong reduction in winter precipitation over the southern part of Europe (Fig. 2c). The resulting precipitation anomaly that occurs between northern and southern Europe during the winter months is also recognized in instrumental records across these regions^1^. This regional decrease in winter precipitation that occurs at the end of the 1970’s is demarcated by the most significant decrease in δ^15^N values of MC guano. Given the direct link between precipitation and the current phase of the NAO, we interpret the δ^15^N values to largely reflect a hydrologic component of the regional climate with a signal that can be related to the NAO.

**Figure 3:**
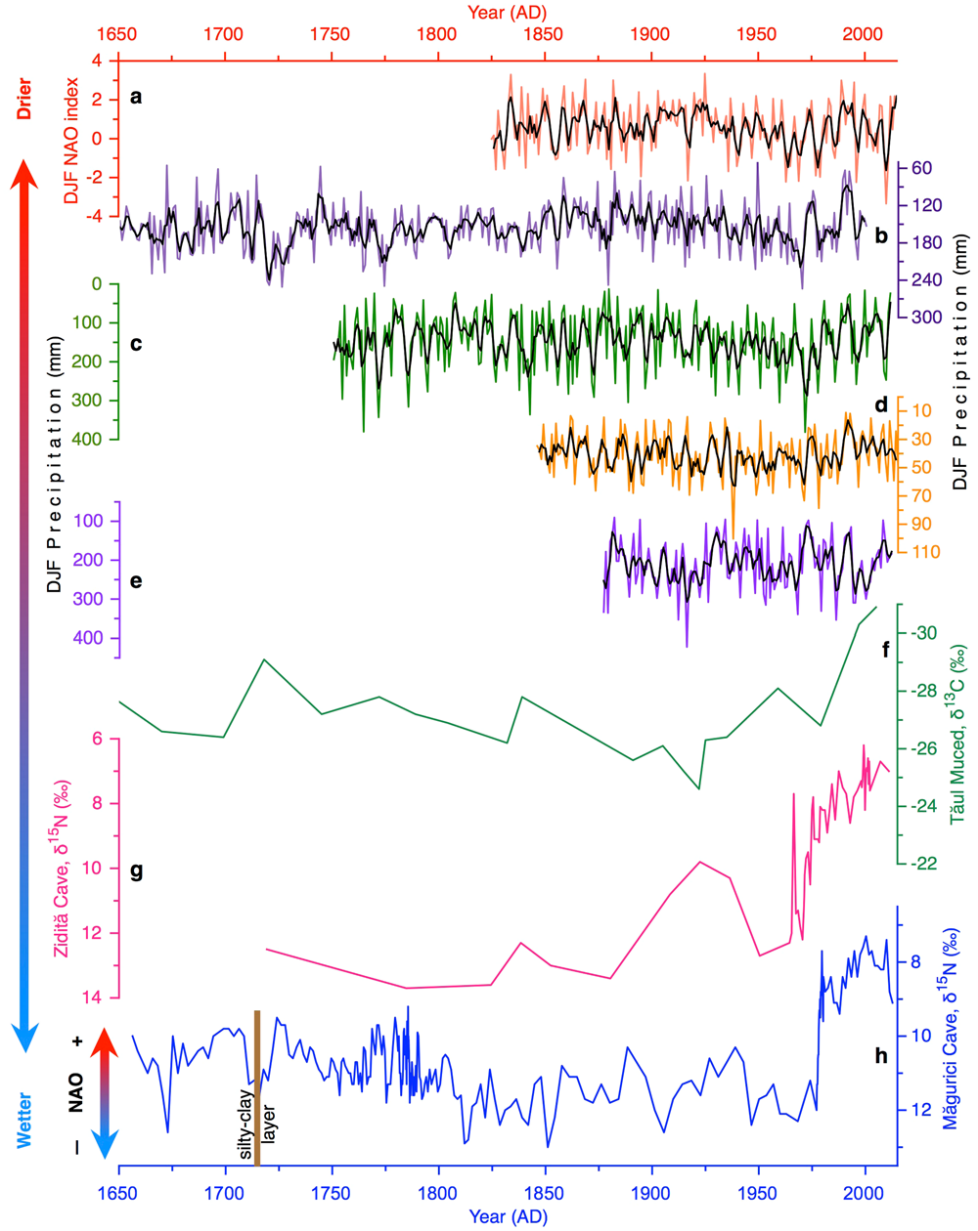
Comparison of guano δ^15^N with other hydroclimate proxy records between AD 1650 and 2012: (**a**) Winter (DJF) NAO index^56^; DJF precipitation data series from ECE^12^ (**b**), Marseille^45^ (**c**), Budapest^45^ (**d**), and Baia Mare^45^ (**e**), δ^13^C of *Sphagnum* from Tăul Muced^46^ (**f**), δ^15^N values of guano from Zidită Cave^26^ (**g**), and Măgurici Cave (**h**; this study). The black smoothed lines in **a–e** represent the 3-year running mean.

Due to the absence of δ^15^N values for certain years it is difficult to confidently utilize a statistical analysis to compare the record directly to the NAO index. However, there is a correlation (p-value = <0.002; R^2^ = 0.43) between the first derivatives of time series of the NAO index and of the δ^15^N values of MC guano (AD 1981–2012; interval of near annual ages of guano; see Supplementary Fig. S3). Additionally, since AD 1800, lower (higher) δ^15^N values, which are indicative of drier (wetter) conditions, occur preponderantly during positive (negative) phases of the NAO (Fig. 3). The occurrence of more negative phases of the NAO (AD 1940–1975) corresponds with progressively wetter conditions expressed in the δ^15^N record. Likewise, trends towards drier conditions interpreted from δ^15^N values appear near contemporaneous with a higher frequency of NAO positive phases (AD 1848–1852; AD 1875–1930; and AD 1975–2012).

Our interpretation of δ^15^N values of MC guano as a NAO proxy (Fig. 3a) is supported by comparison to other precipitation proxies that are more directly influenced by the NAO. Lower δ^15^N values from MC correspond well with drier conditions indicated by a DJF reconstructed precipitation record for ECE^12^ (Fig. 3b), a long-term precipitation measurement in southern France^45^ (Marseille; Fig. 3c), and nearby Budapest and Baia Mare meteorological stations^45^ (Fig. 3d,e). All three are located over regions strongly influenced by NAO (see the blue shaded band in Fig. 1a). Frequently there is contemporaneous overlap between a negative NAO index and wetter conditions indicated by precipitation records and the δ^15^N values from MC guano (Fig. 3h). This agrees with an interpretation of a wetter NW Romania during the negative phase of the NAO. Thus, the state of the N-cycle that is preserved in δ^15^N values of MC guano appears to be influenced by DJF precipitation, which in turn is modulated in ECE by the NAO.

A few of the general trends in hydroclimate that are expressed by the NAO index, meteorological records, and the nitrogen isotopic composition of MC guano can also be found in other Romanian and southern Europe paleo-records spanning the period AD 1850 to present. δ^15^N values from the MC core trend towards wet conditions until AD 1800, fluctuates between 1800 and 1975, after which the climate abruptly became drier until present. Broadly, the δ^15^N values from Zidită and Măgurici guano frequently show similar trends. The two records are well-correlated after AD 1900, when both feature a gradual trend towards drier conditions interrupted by a wetter interval between AD 1940 and 1975 (Fig. 3g,h). δ^15^N values from Zidită, Măgurici, and δ^13^C values in a *Sphagnum* core from the Tăul Muced^46^ (Rodnei Mountains, N. Romania; see Supplementary Fig. S1), all reflect a drying trend after ~AD 1975 (Fig. 3f–h). However, the MC record appears to correspond more consistently with the NAO index, suggesting that the influence of the NAO is more prevalent in NW Romania than in other regions of the Carpathians.

Given that the signal of the NAO instrumental record is reflected in the δ^15^N values since AD 1850, we extend our interpretation of this proxy to infer past phases of the NAO prior to the instrumental record. Indeed, using the ECE DJF precipitation reconstruction^12^ and measurements^45^ (Marseille) as additional evidence, δ^15^N values suggest that the positive phase of the winter NAO dominated the circulation between AD 1650 and 1800. Over this 150-year interval, there were five prominent periods of at least 2 years each during which the negative phase was dominant (Fig. 3h). Our core reveals a major depositional hiatus, when guano accumulation ceased and a silty-clay layer was deposited between ~AD 1713 and 1715 (Fig. 3h). This period corroborates well with one of the highest reconstructed value of DJF precipitation across ECE^12^, suggesting unusually wet winter seasons. Concurrent, historical hydroclimate records elsewhere in Europe^47^ document an increased in flooding frequency at this time. Interestingly, a testate amoeba record from Tăul Muced ombrotrophic bog located ~100 km NE of our cave documents a decline in water table depth over this period^46^. Furthermore, the sharp decrease of the δ^13^C values of *Sphagnum* at the same location (Fig. 3f) was interpreted to indicate prevalence of drier conditions^44^. These site-specific contrasting precipitation patterns are not surprising since the winter NAO signal is stronger in the intra-Carpathian region^8^.

Following the guano hiatus, the δ^15^N time series suggests that beginning at ~AD 1720, a gradual transition to recurring positive phases of the NAO occurred, with the atmosphere remaining locked into this mode until AD 1790. During this 70-year interval, significant negative phases of the pattern appeared only three times (AD 1760, 1775, and 1785), all coincident with wettest winters recorded at the Marseille weather station (Fig. 3c,h). From AD 1800 to 1820, we identified an abrupt transition to more positive nitrogen isotopic values, which suggest increased regional moisture delivery mostly over the winter period. As inferred from our guano δ^15^N values, this begins a period of ~20 years of mild and wet winters in the ECE relative to the earlier interval, which ends ~AD 1840 when precipitation started to decrease. The onset of this period of positive winter NAO phase is coeval with a marked phase of ice ablation in the St. Livre Cave (SW Switzerland), thought to represent almost three decades of warm and dry winters^48^. It also agrees well (but opposite sign) with a composite speleothem annual growth-rate record in NW Scotland, reflective of positive winter NAO states^49^. It is apparent from the discussion above that the changes in the phases of the NAO may partly explain the climate variability over the last part of the LIA.

## Conclusions

A nitrogen isotopic proxy record of guano provides new information regarding the effect of DJF hydroclimate system on the N-cycle and the influence of the winter NAO on the ECE. The evidence of a NAO signal contained within the MC guano δ^15^N series is the temporal strong correlation of the winter precipitation amount in the instrumental record. This study demonstrates that future guano research should consider not only precipitation, but also larger scale climatic systems when utilizing the nitrogen isotopic composition of guano. The use of nitrogen isotopic composition of bat guano it is possible to add to, and improve the historical record of the NAO. As such, our results suggest that the δ^15^N values of guano can be utilized to reconstruct past phases of the NAO beyond the instrumental record and demonstrates that the δ^15^N values of guano can offer a proxy of the NAO in regions where instrumental or historical records are limited.

## Methods

### Guano coring and sampling

In October 2012, a Russian peat corer was used to extract a 287 cm core from a guano pile located in the Circular Room of MC (Fig. 1 in Johnston *et al*.^50^). The coring site was adjacent (within 1 m) to the one investigated by Johnston *et al*.^50^. Both cores have similar stratigraphy, however, the lower 29 cm of the first one recovered only clay, whereas ours penetrated a 4-cm thick clay layer at 237 cm revealing an additional 46 cm of guano beneath it. Except for the clay layer, the entire core length was sampled for isotopic analyses and radiocarbon measurements.

### Radiocarbon dating and age-depth models

Twenty aliquots of bulk bat guano from various depths of the Măgurici core were submitted for radiocarbon dating by accelerator mass spectrometry (AMS) at the Poznan Radiocarbon Laboratory (Poland) and returned ages in stratigraphic order. Sample MG-15 was contaminated with young organic matter and thus discarded. Since guano is mainly composed of chitin (>95%), which makes it an excellent material for AMS age determinations^51^, no sample preparation was needed.

The age-depth models (see Supplementary Fig. S4) are based on a linear interpolation between each^14^C age in the upper 50 cm of the core, whereas for the rest of the sequence a type 4 smooth spline was applied. Both models were generated using Clam code^52^. The reasoning for employing a linear age-depth model for the upper 50 cm is because continuous observations since 1965 confirmed that the size of the bat colony has not changed, and therefore, it is expected that the guano accumulation remained constant. The default calibration curve utilized by Clam is the northern hemisphere terrestrial curve IntCal13.14C (cc = 1) from Reimer *et al*.^53^. The samples in the top 50 cm of the core are characterized by high radiocarbon activity (130.06 ± 0.4 and 132.46 ± 0.34 pMC) resulting in modern ages (1979–1980 and 1977–1978 cal. years). Guano began to accumulate in the Circular Room at ~AD 881, shortly before the beginning of the Medieval Warm Period (MWP: ~AD 950–1300). One hiatus is inferred from the age depth model between AD 1237–1651, an interval that corresponds to the first half of the LIA. The raw^14^C data are included in Supplementary Dataset 2, and the results of modeling in Supplementary Dataset 3).

### Elemental and stable isotope analyses

Contiguous 1-cm bulk guano sub-samples were recovered for isotopic analyses along with a modern sample collected in 2012 to anchor the isotope chronology. Chitin is the dominant organic compound in MC guano, therefore, we considered compound specific extraction to be unnecessary. Due to the cave climate (see Supplementary Information) it is highly unlikely that any soluble guano-derived N-compound will survive and potentially impact the nitrogen isotopic composition.

All samples were prepared for δ^15^N and δ^13^C analysis following the procedures described in Forray *et al*.^25^ and Cleary *et al*.^26^. Out of these samples, 1–2 mg aliquots were weighed and placed in tin cups and then measured for δ^15^N, δ^13^C, %N, %C, and C:N. Analysis was completed using a Costech Elemental Analyzer coupled to a Delta V Isotope Ratio Mass Spectrometer hosted in the Stable Isotope Laboratory (School of Geosciences, University of South Florida). A glutamic acid (internal standard; δ^15^N: −6.28‰; δ^13^C: −16.50‰; %N: 9.54%; %C: 41.37%) and a protein standard B2155 (δ^15^N: 5.94‰; δ^13^C: −26.98‰; %N: 13.32%; %C: 46.5%) were used during analysis. Certified reference materials, B2155 and IAEA-N1, were used to calibrate the δ^15^N value for the internal standard. B2155 and IAEA-C7 were used to calibrate δ^13^C value of the glutamic acid. Estimation of the precision of analysis (δ^15^N: 0.08‰; δ^13^C: 0.04‰) was based on replicate internal standards during each run (Supplementary Dataset 1).

### Statistical methods

Correlation analysis between δ^15^N values (mean = 10.4‰; std. dev. = 1.5) and δ^13^C values (mean = −24.5‰; std. dev. = 0.7) (n = 105) was completed using SPSS. This statistical test is appropriate as both data sets were extracted from the MC core with the same sampling and temporal resolution. Resulting p value andR^2^ (p-value = <0.001; R^2^ = 0.62) from the 2-tailed test indicate statistical correlation. MATLAB was used to convert unevenly sampled data to evenly sampled δ^15^N values, whereas EXCEL was used to compute the first derivatives (in 1-year time steps). This step allowed for the examination of year-to-year changes in values. Correlation analysis (n = 26; using SPSS) between the first derivatives of δ^15^N time series (mean = 8.4‰; std. dev. = 0.6) and DJF NAO index (mean = 0.5; std. dev. = 1.4) data sets was completed. This analysis was performed for years between 1981 and 2012 that included a respective δ^15^N value. The decision of testing the derivatives is appropriate due to the fact that the sensitivity of change being a more probable representation of influence of the NAO then raw values. The 2-tailed test resulted in a p-value = <0.002 and R^2^ = 0.43.

## Electronic supplementary material

Supplementary information

Dataset 1 (table)

Dataset 2 (table)

Dataset 3
