# Supplementary material for: Evidence of long-term NAO influence on East-Central Europe winter precipitation from a guano-derived δ15N record
Source: Sci Rep. 2017 Oct 26;7:14095. doi: 10.1038/s41598-017-14488-5 (PMC5658435; doi:10.1038/s41598-017-14488-5)
Supplement: Supplementary file 1 — Supplementary information [file 41598_2017_14488_MOESM1_ESM.docx]

**Evidence of long-term NAO influence on East-Central Europe winter precipitation from a guano-derived** δ**^15^N record**

Daniel M. Cleary^1^, Jonathan G. Wynn^1,2^, Monica Ionita^3,4^, Ferenc L. Forray^5^, Bogdan P. Onac^1,5,6^*

^1^School of Geosciences, University of South Florida, 4202 E. Fowler Ave., NES 107, Tampa, FL 33620, USA.

^2^Current Address: National Science Foundation, 4201 Wilson Blvd., Arlington, VA 22230, USA.

^3^Paleoclimate Dynamics Group, Alfred-Wegener-Institute for Polar and Marine Research, Bussestrasse 24, Bremerhaven, D-27570, Germany.

^4^MARUM, Center for Marine Environmental Sciences, University of Bremen, Germany.

^5^Department of Geology, Babeș-Bolyai University, Kogălniceanu 1, 400084 Cluj-Napoca, Romania.

^6^Emil Racoviță Institute of Speleology, Romanian Academy, Clinicilor 5, Cluj-Napoca, 400006, Romania.

*Correspondence and requests for materials should be addressed to BPO (bonac@usf.edu)

**Supplementary Information**

**Study site.** Măgurici Cave is located east of the Răstoci village (Someș Plateau) in NW Romania^1^, at less than 1 km from the Someș River (see Supplementary Fig. S1b). It is a 543 m long cave developed in a thick (40-60 m) Eocene/Oligocene fossiliferous limestone^2-4^. The landscape around the cave consists of low elevation rolling hills separated one from another by dry valleys, small karst depression, and alignments of dolines. Except some patches of forest composed of *Fagus sylvatica*, *Quercus petrea*, *Carpinus betulus*, *Populus*, and *Salix* (all C_3_-type plants), most of the terrain is represented by meadows, shrubland, and limited arable fields. Anthropogenic impacts include small-scale deforestation and agriculture activities^5^.

The annual average temperature in the Răstoci region is 9.5ºC, whereas in the Măgurici’s Cave Circular Room from where the guano core was recovered, the average is slightly higher (10.9ºC) and the relative humidity remains above 95% all year round^6^. Mean annual precipitation on this part of the Someș Plateau reaches 583 mm, of which 388 mm are recorded during winter and spring^7^.

Large guano accumulations are known from three areas of the Măgurici Cave: the Suspended Room, the Guano Gallery, and the Circular Room (for locations see Fig. 1 of Johnston *et al*^8^). The dominant bat species are *Myotis myotis*, *Myotis blythii*, and *Minopterus scheibersii*, but the first two only gather between April and end of August in large maternity colonies (hundreds to one thousand bats)^6^*. M. myotis* forage ~5 km from their roosting site over deciduous woodlands. *M. blythii* are often found in mixed colonies with *M. myotis*, but more commonly forage in grassland habitats.

**Supplementary References**

1. Onac, B. P. & Todoran, V. Contribution à la connaissance des formations de gypse de la grotte de Răstoci (NO de la Roumanie). In *The Eocene from the Transylvanian Basin* (eds Petrescu, I. *et al*.) 301-306 (University Cluj-Napoca, 1987).
2. Bucur, I. Ι., Onac, B. & Todoran, V. Algues calcaires dans les dépôts oligocènes inférieurs de la région Purcăreţ - Mesteacăn - Valea Chioarului (NW du Basin de Transylvanie). In *The Oligecene from the Transylvanian Basin* (eds Petrescu, I. *et al*.) 141-148 (University Cluj-Napoca, 1989).
3. Todoran, V. & Onac, B. P. Eocene/Oligocene boundary in the Purcăreț-Mesteacăn area (The Someș Plateau). In *The Oligocene from the Transylvanian Basin* (eds Petrescu, I. *et al*.) 129-132 (University Cluj-Napoca, 1989).
4. Prică, I. Coralgal facies of the Upper Eocene–Lower Oligocene limestones in Letca–Răstoci Area. S*tudia UBB Geologia* **46,** 53-61 (2001).
5. Geantă, A, Tanțău, I., Tămaș, T. & Johnston, V. E. Palaeoenvironmental information from the palynology of an 800 year old bat guano deposit from Măgurici Cave, NW Transylvania (Romania). *Rev*. *Palaeobot. Palynol.* **174**, 57**-**66 doi:http://dx.doi.org/10.1016/j.revpalbo.2011.12.009 (2012).
6. Borda, D., Borda, C. & Tămaș, T. Bats, climate, and air microorganisms in a Romanian cave. Ma*mmalia* **68,** 337-343 doi:<https://doi.org/10.1515/mamm.2004.033> (2004).
7. Sandu, I., Pescaru, V. I. & Poiană, I. (eds) *The climate of Romania* (Ed. Acad. Rom., 2008).
8. Johnston, V. E., McDermott, F. & Tămaș, T. A radiocarbon dated bat guano deposit from N.W. Romania: implications for the timing of the Little Ice Age and Medieval Climate Anomaly. Pa*laeogeogr. Palaeoclimatol. Palaeoecol.* **291,** 217- 227 doi:doi.org/10.1016/j.palaeo.2010.02.03 (2010).
9. Cleary, D. M., Onac, B. P., Forray, F. L. & Wynn, J. G. Effect of diet, anthropogenic activity, and climate on δ^15^N values of cave bat guano. *Palaeogeogr. Palaeoclimatol. Palaeoecol*. **461**, 87-97 doi:doi.org/10.1016/j.palaeo.2016.08.012 (2016).
10. Feurdean, A. *et al*. Last millennium hydro-climate variability in Central-Eastern Europe (Northern Carpathians, Romania). *The Holocene* **25**, 1179-1192 doi:doi.org/10.1177/0959683615580197 (2015).


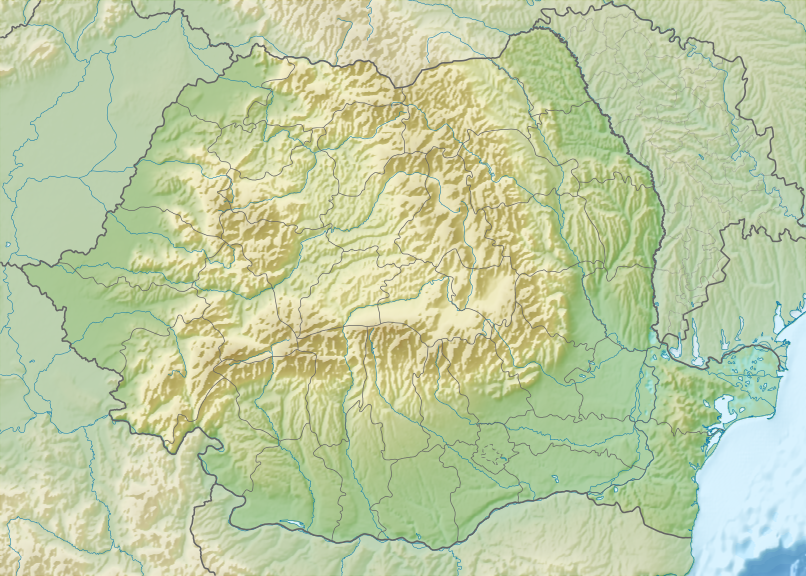


Cluj-Napoca

Bucharest


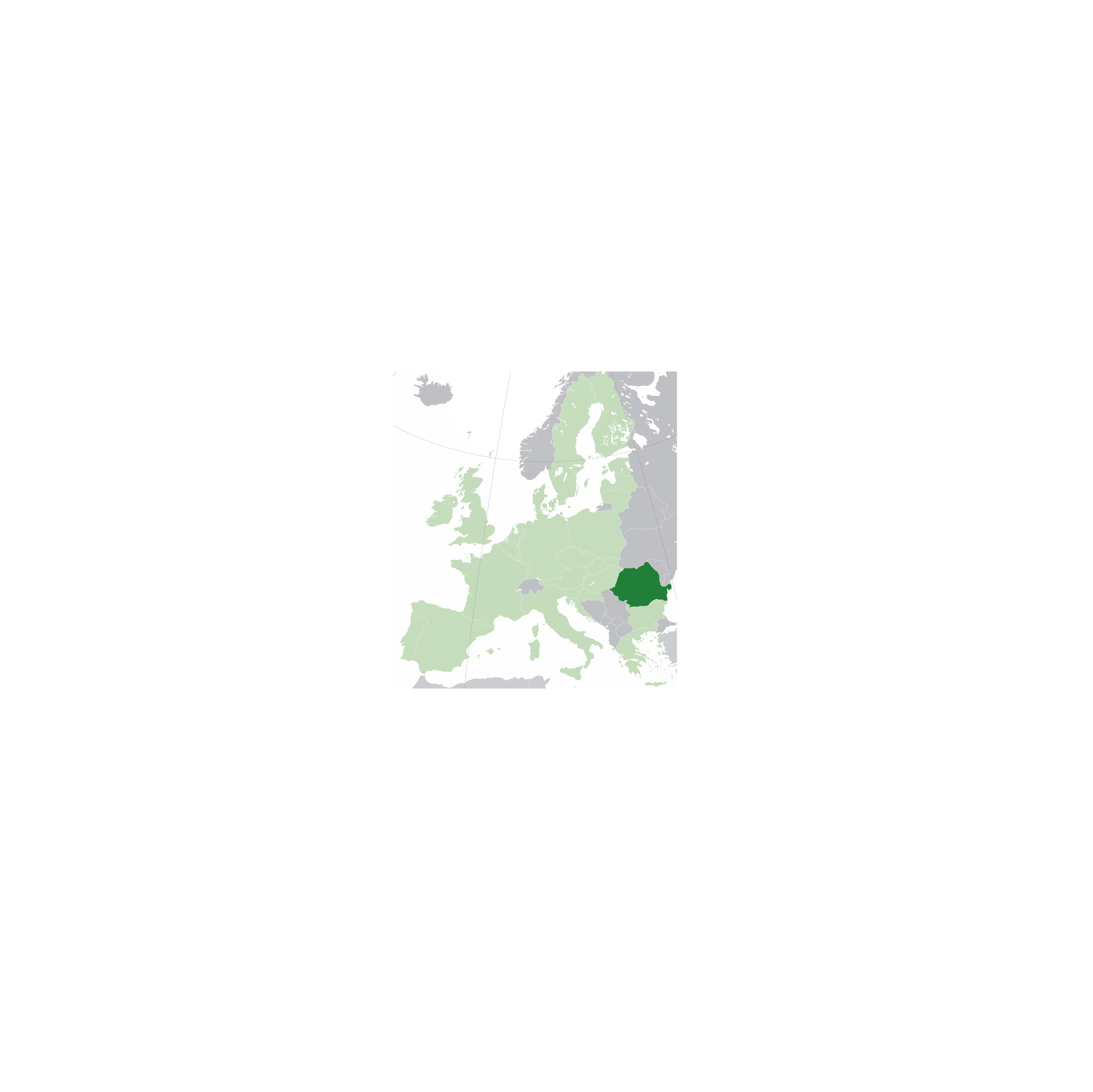


Marseille

Budapest

Baia-Mare

**Figure S1.** Study area in the East-Central Europe. (**a**) Map outlines Romania (dark green filled) and the location of weather stations mentioned in text. (**b**) Elevation map of Romania showing Măgurici Cave (white star) and other sites discussed in text; Zidită Cave^9^ (white dot), Tăul Muced^10^ (turquoise dot), and the Baia Mare Weather Station (yellow dot). Maps of Europe and Romania are available under CC BY-SA 3.0 license (https://creativecommons.org/licenses/by-sa/3.0/) from https://commons.wikimedia.org/wiki/File:EU-Romania.svg and https://upload.wikimedia.org/wikipedia/commons/5/59/Relief_Map_of_Romania.png, respectively.
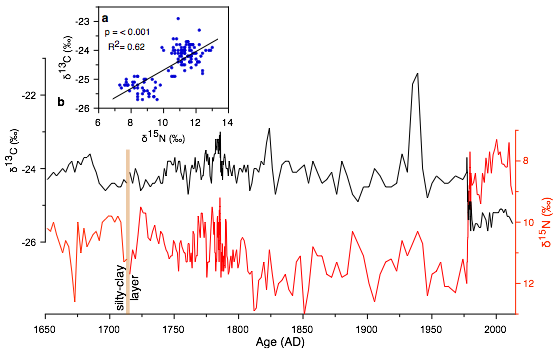


**Figure S2.** (**a**) Correlation analysis between δ^15^N and δ^13^C values of Măgurici Cave guano from AD 1800 to 2012. (**b**) δ^15^N (red) and δ^13^C (black) values of MC guano from AD 1650 to 2012. The silty-clay layer interbedded in the guano deposit between 237 and 241 cm in depth is shown by a brown bar.

**Figure S3.** Correlation between the derivatives of time series of δ^15^N
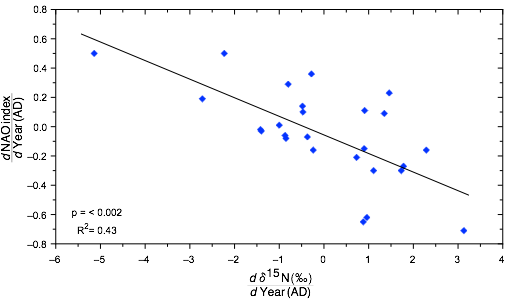
values of Măgurici Cave guano and DFJ NAO index from AD 1981 to 2012.


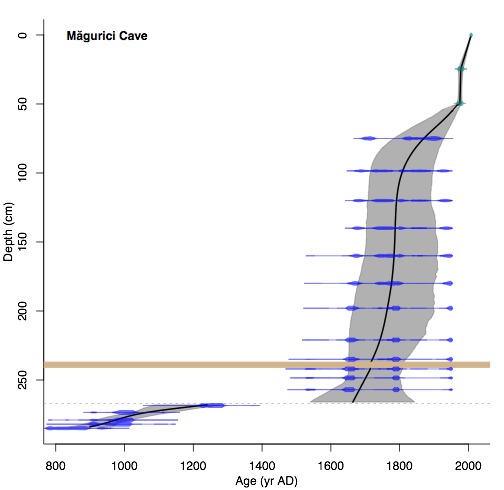


**Figure S4.** Age-depth model for Măgurici guano core, showing the position of the silty-clay layer (brown bar).
